# Target trial emulation of statin discontinuation in multimorbid older adults with polypharmacy

**DOI:** 10.1111/eci.70126

**Published:** 2025-09-30

**Authors:** Valerie Aponte Ribero, Oliver Baretella, Cinzia Del Giovane, Moa Haller, Martin Feller, Benoît Boland, Antoine Christiaens, Wilma Knol, Denis O'Mahony, Viktoria Gastens, Baris Gencer, Stéphanie Baggio, Nicolas Rodondi

**Affiliations:** ^1^ Institute of Primary Health Care (BIHAM), University of Bern Bern Switzerland; ^2^ Graduate School for Health Sciences University of Bern Bern Switzerland; ^3^ Department of General Internal Medicine Inselspital, Bern University Hospital Bern Switzerland; ^4^ Department of Medical and Surgical Sciences for Children and Adults University of Modena and Reggio Emilia Modena Italy; ^5^ Department of Geriatric Medicine, Cliniques Universitaires Saint‐Luc Université Catholique de Louvain Brussels Belgium; ^6^ Clinical Pharmacy Research Group Louvain Drug Research Institute (LDRI), Université Catholique de Louvain Brussels Belgium; ^7^ Fonds de la Recherche Scientifique – FNRS Brussels Belgium; ^8^ Department of Geriatrics and Expertise Centre Pharmacotherapy in Old Persons (EPHOR) University Medical Center Utrecht, Utrecht University Utrecht the Netherlands; ^9^ Department of Medicine, School of Medicine University College Cork, National University of Ireland Munster Ireland; ^10^ Department of Geriatric Medicine Cork Cork University Hospital Group Munster Ireland; ^11^ Population Health Laboratory #PopHealthLab University of Fribourg Fribourg Switzerland; ^12^ Cardiology Division Geneva University Hospitals Geneva Switzerland; ^13^ Department of Cardiology Lausanne University Hospitals Lausanne Switzerland; ^14^ Institute of Psychology University of Lausanne Lausanne Switzerland

**Keywords:** cardiovascular disease, mortality, statins

## Abstract

**Background:**

The benefit of statins in multimorbid older adults is controversial. Prior observational studies evaluating statin discontinuation in older adults were retrospective cohorts, did not focus on multimorbidity, or lacked adjustment for geriatric syndromes. We aimed to assess the effect of statin discontinuation on cardiovascular and mortality outcomes using the target trial emulation framework.

**Methods:**

We conducted a prospective cohort study using data from the OPERAM trial in adults aged ≥70 years with ≥3 chronic conditions and ≥5 chronic drugs, comparing statin discontinuation to continuation. The primary composite outcome was cardiovascular events or all‐cause mortality at 12 months. We calculated adjusted hazard ratios (HR) using weighted pooled logistic regressions without (model‐A) and with adjustment for two geriatric syndromes (falls and weight loss; model‐B).

**Results:**

Of 2668 person‐trial units (mean age 78.5 years), 2533 (95%) continued and 133 (5%) discontinued statins. Discontinuation was associated with higher composite outcome risk (27% vs. 18%; HR model‐A 1.53 [95% CI 1.14–2.06]; model‐B 1.49 [1.12–1.99]). This was mainly attributable to increased non‐cardiovascular deaths (20% vs. 11%; HR model‐A 1.56 [1.08–2.27]; model‐B 1.52 [1.06–2.19]); there was no clear evidence for an association with cardiovascular events (7% vs. 8%; HR model‐A 1.36 [.86–2.14]; model‐B 1.35 [.86–2.12]).

**Conclusion:**

In this first target trial emulation in a multimorbid older population, statin discontinuation was associated with increased risk of the composite of cardiovascular events or all‐cause mortality, primarily driven by non‐cardiovascular deaths. Geriatric syndromes did not modify these increased risks. Only clinical trials can clarify the safety of statin discontinuation.

## Graphical abstract

In this target trial emulation in multimorbid older adults with polypharmacy, statin discontinuation was associated with a higher rate of non‐cardiovascular mortality, suggesting residual confounding by indication. Unlike some previous retrospective cohort studies, there was no clear evidence of increased cardiovascular events arising from statin discontinuation in this patient cohort. Adjusting for two geriatric syndromes (falls and weight loss) did not change results. Only data from dedicated clinical trials can clarify the safety of statin discontinuation in multimorbid older adults.

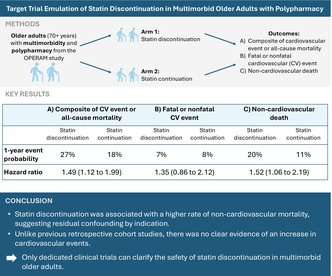

## 1 INTRODUCTION

Statin use is highly prevalent among older persons aged ≥70 years, with prevalence ranging from 15% to 70%.^1^ However, robust evidence from clinical trials on the benefits of statins for primary cardiovascular prevention in older adults with multimorbidity is lacking.^2^, ^3^ Benefits of statins are well established in secondary prevention but with limited data in multimorbid populations due to their underrepresentation in clinical trials.^4^ Moreover, older adults with multimorbidity and polypharmacy are at increased risk of statin side effects.^2^ Consequently, statin deprescribing may be considered in this population, according to guidelines of the American Heart Association.^5^ However, clear evidence from randomized controlled trials (RCT) on the effects of statin discontinuation in multimorbid older people is sparse. One small RCT in patients in palliative care reported improved quality of life with no increase in cardiovascular events after statin discontinuation.^3^ However, this was a highly selected patient cohort with poor short‐term survival prognosis. A pragmatic primary prevention RCT in older patients with multimorbidity is currently ongoing, but results will not be expected before 2026.^6^

Target trial emulation as a framework for observational data analyses has the potential to provide valid causal estimates using observational data when data from RCTs are unavailable.^7^ Three observational studies in older adults found an increased risk of cardiovascular events, and one found no difference in all‐cause mortality after statin discontinuation prescribed for primary and secondary prevention.^8^, ^9^, ^10^, ^11^ However, all were retrospective cohort studies, which may limit the accuracy of data collection; none specifically examined a multimorbid population, and some did not adjust for geriatric syndromes, which may be confounding factors as they are associated with adverse medication effects and poor outcomes.^8^, ^9^, ^10^, ^12^, ^13^ Furthermore, none of these studies used a clone‐censor‐weight approach to handle immortal‐time bias, which has been recommended as better than other approaches as it avoids selection bias and offers a clearer interpretation of the estimand.^14^

In this study, we employed the target trial emulation framework to estimate the effect of statin discontinuation versus continuation on clinical outcomes in a well‐characterized prospective cohort of multimorbid older adults with polypharmacy using data from a recent large‐scale multicenter clinical trial.^15^ The primary objective was to assess the impact of statin discontinuation on cardiovascular events and mortality outcomes using two models with and without adjustment for geriatric syndromes. Secondary objectives were to assess the effect of statin discontinuation on falls, activities of daily living functionality and quality of life.

## 2 METHODS

### 2.1 Data source

Data for this target trial emulation was obtained from the OPERAM clinical trial (Optimizing thERapy to prevent Avoidable hospital admissions in Multimorbid older adults).^15^ The trial enrolled 2008 hospitalized patients aged 70 years or older with multimorbidity (≥3 chronic conditions) and polypharmacy (≥5 drugs used for over 30 days prior to eligibility assessment) in four European countries (Belgium, Ireland, the Netherlands and Switzerland) and enrolled patients from December 2016 to October 2018. The trial objective was to assess the effect of a medication review intervention versus usual pharmaceutical care on the first drug‐related hospital admission within 12 months after hospital discharge.^15^ Detailed data on medication use were collected at enrolment and follow‐up visits post‐randomization. Ethics committees at each site approved the trial and written informed consent was obtained from each participant.

### 2.2 Participants

Participants aged ≥70 years with multimorbidity and polypharmacy who were taking statins at enrolment were eligible for inclusion in the current study. From a total of 1115 eligible patients under statins, 38 patients (3%) were excluded due to missing follow‐up data after discharge, 36 patients (3%) were excluded due to missing covariate data, and 13 patients (1%) were excluded as they had discontinued statins over 15 days prior to hospital discharge (Figure S1). Overall, 1028 statin‐treated patients were included in the current analysis.

### 2.3 Exposure

We compared statin discontinuation to statin continuation within three sequential emulated trials starting at hospital discharge, 2 months and 6 months follow‐up to maximize the number of participants (Figure S2). All enrolled patients were followed for 12 months post‐enrolment. Patients were assigned to the statin discontinuation or continuation group at each of these timepoints, based on medication data collected at discharge, 2‐month and 6‐month follow‐up from at least one of the following sources: patients (or proxies), general practitioners, medical charts and/or charts from the local pharmacy. Statins were identified using the Anatomical Therapeutic Chemical (ATC) code C10AA. Since the recorded date of statin discontinuation did not always align with the follow‐up dates, we adopted a grace period of 30 days^16^ around the date of each starting timepoint (discharge, 2 months and 6 months follow‐up) to assign participants to statin discontinuation or continuation (Figure S2), and used a ‘clone‐censor‐weight’ approach to avoid any resulting immortal‐time bias.^14^ Although the majority of statin discontinuations were captured using the 30‐day grace period, to account for any potential misclassification of participants discontinuing statins afterwards, we conducted a sensitivity analysis prolonging the grace period to 3 months. Furthermore, we conducted a per‐protocol analysis in which participants were censored when restarting statins in the discontinuation group, or when discontinuing statins in the continuation group.

### 2.4 Outcomes

The primary outcome was a composite of first occurrence of a cardiovascular event (a composite of hospitalization for acute coronary syndrome, stroke, transient ischemic attack, or peripheral vascular disease; these are atherosclerotic cardiovascular outcomes as defined by the American Heart Association^17^) or all‐cause death within 12 months. All‐cause death (and not cardiovascular death only) was selected in the composite to account for competing mortality among this multimorbid older population and a possible shift from cardiovascular to other causes of death (such patterns were seen in two statin RCTs).^18^ Cardiovascular events were identified via International Classification of Diseases, Tenth Revision (ICD‐10) diagnoses of hospital admissions (Table S1) and via serious adverse events reported as ‘acute coronary syndrome’ or ‘stroke’ which were reported for all patients as part of the OPERAM trial. Follow‐up of participants started at hospital discharge, 2 months or 6 months, respectively, and ended at the first occurrence of the outcome, death, loss to follow‐up, or end of follow‐up (12 months; Figure S2). We also evaluated the components of the composite outcome incorporating (i) first fatal or non‐fatal cardiovascular event and (ii) non‐cardiovascular death. Secondary outcomes included incident falls, health‐related quality of life (measured using the EQ‐5D visual analogue scale [VAS]) and functional independence (measured using Barthel index; dichotomized using a cutoff score of 90 [corresponding to none or slight vs. moderate to total dependence]^19^ due to severe skewness) at 12 months. Different cut‐offs for the Barthel index were explored in sensitivity analyses. As a negative control outcome, we assessed the impact of statin discontinuation on hospitalization for gastrointestinal disorders to assess the potential for residual confounding due to healthy‐user effects.

### 2.5 Statistical analysis

As this was a secondary analysis, no sample size was calculated a priori. We performed a sensitivity power analysis using G*Power 3.1 to determine the minimum effect size the study could detect. With alpha = .05, power = .80, *n* = 2533 in the no statin group, *n* = 133 in the statin group, and a two‐tailed independent t‐test, we could detect an effect size of *d* = .25. Therefore, our study was powered to detect medium effect sizes.

The specifications of the target trial emulation are available in Table S2. We presented unweighted crude baseline characteristics and effect estimates using the pre‐cloned dataset. Baseline characteristics are shown in person‐trial units, which reflect the number of unique trial entries rather than unique individuals. In target trial emulation, patients can contribute to multiple trials (e.g., a person on statins may be eligible for the statin continuation group at multiple time points) and are therefore counted separately for each trial entry.

We used a three‐step ‘clone‐censor‐weight’ approach for the emulation.^14^ First, all patients were cloned and assigned to each treatment group (statin discontinuation and statin continuation). Since each group comprised all included patients, the groups were balanced in terms of all (measured and unmeasured) baseline characteristics by definition. Second, each clone was censored once they deviated from their assigned treatment group. A clone assigned to the statin discontinuation group was censored at the end of the grace period, while a clone assigned to the statin continuation group was censored once they discontinued the statin during the grace period. Third, patients were weighted using time‐varying inverse probability of censoring weights (IPCW) to account for selection bias from censoring and loss to follow‐up. Our assumed confounding structure is presented in Figure S3. Two logistic regression models were fitted to calculate stabilized IPCW for each emulated trial separately: model A was fitted with treatment group (discontinuation vs. continuation) as the dependent variable and time from baseline (in days), OPERAM randomization group, study site, age, sex, statin intensity (low/moderate vs. high),^17^ use of glucose‐lowering drugs (ATC code A10), presence of comorbidities (cardiovascular disease, cancer, chronic respiratory disease, heart failure; Table S1) and Barthel index (≥90 vs. <90) as independent variables (Figure S2). Model B additionally included two geriatric syndromes, number of falls in the previous year (0, 1, 2 or 3+) and weight loss in the previous year (yes or no), as independent variables.

Outcomes were analysed with pooled data from all three emulated trials. For time‐to‐event outcomes, we estimated hazard ratios (HR) and 95% confidence intervals (CI) using IPCW‐weighted pooled logistic regression models, which approximate a time‐dependent Cox model when time intervals are short (1 day in our study).^20^ A post‐hoc decision was made to present weighted cumulative incidence curves and 12‐month event probabilities (Aalen‐Johansen estimates) for the primary outcome, following recommendations to report absolute risks in addition to hazard ratios.^21^ We estimated odds ratios (OR) for binary outcomes using weighted logistic regressions and mean differences for continuous outcomes using weighted linear regressions. Robust standard errors were calculated to account for the fact that patients may be included multiple times across the three emulated trials.

We performed stratified analyses by primary and secondary cardiovascular prevention and computed interaction p‐values by adding an interaction term between treatment group and history of cardiovascular disease to model B. We conducted several sensitivity analyses: running the analyses without a grace period and lengthening it to 3 months; including new cancer diagnoses as a time‐varying confounder; including hospitalizations for heart failure in the cardiovascular event definition; using other commonly used cut‐offs of the Barthel index (≤60 for severe dependence, ≤20 for total dependence); and excluding participants with cancer at baseline.^19^ We also conducted a ‘per‐protocol’ analysis in which patients were censored upon deviation from their assigned treatment group (i.e., restarting statins in the discontinuation group and discontinuing statins in the continuation group). For continuous and binary outcomes, those patients were excluded from the ‘per‐protocol’ analyses. Finally, we calculated the E‐value for confounding to assess the magnitude of confounding necessary to explain away the results.^22^ Analyses were conducted using R statistical software, version 4.3.2.

## 3 RESULTS

### 3.1 Cohort characteristics

We included data from a total of 1028 unique patients contributing to 2668 person‐trial units across the three emulated trials (Table 1). The mean age was 78.5 years (standard deviation [SD] 5.9) and 38% were women. A total of 73% of patients had established cardiovascular disease and 31% used high‐intensity statins at baseline. Statins were discontinued within 1 month by 133 (5%) patients while 2535 (95%) patients continued statins. Of those who discontinued statin therapy, 57 patients (43%) did not have prior cardiovascular disease and 76 patients (57%) had established cardiovascular disease. The reason for discontinuation was undocumented in most cases (62%). When a reason was stated, ‘no current indication’ was the most common reason given (22% of cases). Patients in the statin discontinuation group were older (80.2 years vs. 78.5 years), fewer had cardiovascular disease (57.1% vs. 73.3%) and were less functionally independent (Barthel index <90; 51.9% vs. 38.9%) than patients in the continuation group. Patients in Bern (Switzerland) and Louvain (Belgium) were more likely to discontinue statins than patients in Cork (Ireland) and Utrecht (the Netherlands). Other characteristics were similar between groups. Over a median follow‐up of 9.9 months (interquartile range: 6.1–11.7), 395 (14.9%) patients experienced the primary outcome. A total of 163 patients (6.1%) had a fatal or nonfatal cardiovascular event and 249 (9.3%) patients died due to non‐cardiovascular causes. The most common cause of non‐cardiovascular death was disseminated cancer (30% of deaths; Table S3).

**TABLE 1:** Baseline characteristics of OPERAM participants with statin medication (in person‐trial units; pre‐cloned data).

Characteristics	Overall	Statin continuation	Statin discontinuation	*p*‐Value
	(*n* = 2668)	(*n* = 2535; 95%)	(*n* = 133; 5%)	
Socio‐demographics				
Age (years)	78.5 (5.9)	78.5 (5.8)	80.2 (6.9)	.001
Women	1025 (38.4%)	977 (38.5%)	48 (36.1%)	.635
Study sites				
Bern	1065 (39.9%)	1005 (39.6%)	60 (45.1%)	.035
Cork	539 (20.2%)	519 (20.5%)	20 (15.0%)	
Louvain	415 (15.6%)	386 (15.2%)	29 (21.8%)	
Utrecht	649 (24.3%)	625 (24.7%)	24 (18.0%)	
Trial randomization group				
OPERAM Control	1403 (52.6%)	1331 (52.5%)	72 (54.1%)	.781
OPERAM Intervention	1265 (47.4%)	1204 (47.5%)	61 (45.9%)	
Medication				
Number of chronic medications	10 (7–13)	10 (7–13)	10 (7–13)	.757
Statin type				
Atorvastatin	1261 (47.3%)	1192 (47.0%)	69 (51.9%)	.691
Fluvastatin	41 (1.5%)	38 (1.5%)	3 (2.3%)	
Pravastatin	213 (8.0%)	204 (8.0%)	9 (6.8%)	
Rosuvastatin	535 (20.1%)	513 (20.2%)	22 (16.5%)	
Simvastatin	618 (23.2%)	588 (23.2%)	30 (22.6%)	
Statin intensity^a^				
High	826 (31.0%)	791 (31.0%)	40 (30.1%)	.896
Low/moderate	1842 (69.0%)	1749 (69.0%)	93 (69.9%)	
Glucose‐lowering drugs	821 (30.8%)	783 (30.9%)	38 (28.6%)	.640
At least one drug–drug interaction^b^	1557 (58.4%)	1475 (58.2%)^c^	82 (61.7%)	.483
Comorbidities				
Cardiovascular disease	1934 (72.5%)	1858 (73.3%)	76 (57.1%)	<.001
Heart failure	624 (23.4%)	589 (23.2%)	35 (26.3%)	.476
Chronic respiratory disease	1180 (44.2%)	1116 (44.0%)	64 (48.1%)	.402
Cancer (active malignancy except skin)	717 (26.9%)	678 (26.7%)	39 (29.3%)	.580
Health and frailty indicators				
Barthel index^d^ <90	1054 (39.5%)	985 (38.9%)	69 (51.9%)	.004
EQ‐5D VAS^e^	60.2 (20.5)	60.3 (20.5)	58.0 (20.6)	.203
Number of falls in the previous year				
0	1704 (63.9%)	1626 (64.1%)	78 (58.6%)	.623
1	475 (17.8%)	449 (17.7%)	26 (19.5%)	
2	201 (7.5%)	189 (7.5%)	12 (9.0%)	
3+	288 (10.8%)	271 (10.7%)	17 (12.8%)	
Lost weight in the previous year	860 (32.2%)	817 (32.2%)	43 (32.3%)	1.000

*Note*: Numbers are presented as *n* (%), mean (SD) or median (Q1–Q3) as appropriate.

Abbreviations: SD, standard deviation; VAS, visual analogue scale.

^a^ Statin intensity classified according to Stone et al.^17^

^b^ Identified based on a list of 66 potentially clinically relevant drug–drug interactions.^26^

^c^ Of those, *n* = 255 (10%) had a drug–drug interaction that involved a statin (DDI25, DDI26, DDI27, DDI28, DDI29).

^d^ Score to assess activities of daily living; 0 points corresponding to complete dependency, 100 points to complete independency in all domains.

^e^ Questionnaire‐based health status on a 0 (death) to 100 (perfect health) visual analogue scale.

### 3.2 Impact of statin discontinuation on clinical outcomes

**FIGURE 1:**
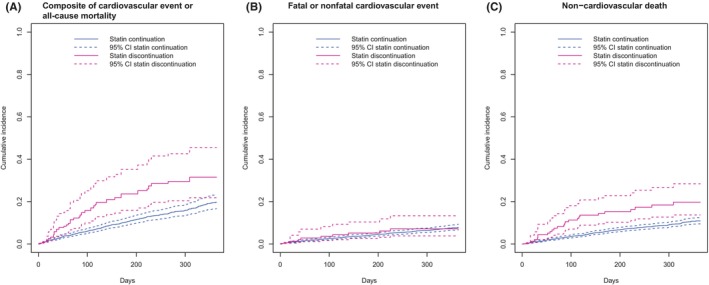
Weighted cumulative incidence curves of death and cardiovascular event outcomes. Weighted cumulative incidence curves were estimated using stabilized inverse probability weights controlling for randomization group, study site, age, sex, glucose‐lowering drugs, statin intensity, Barthel index, comorbidities (cardiovascular disease, cancer [except skin], chronic respiratory disease, heart failure), number of falls and weight lost in the previous year.

Results are shown in Table 2. The crude number of patients who died or had a cardiovascular event at 12 months was 33 (24.8%) in the discontinuation group and 362 (14.3%) in the continuation group, with a crude HR of 1.83 (95% CI 1.28–2.62). After cloning and weighting, the 12‐month event probability was 27% (95% CI 18%–35%) for statin discontinuation and 18% (95% CI 15%–21%) for statin continuation (Figure 1). The rate of the primary outcome remained higher for statin discontinuation versus continuation (HR 1.53, 95% CI 1.14–2.06). Additional adjustment for the number of falls and weight loss in the previous year resulted in a similar HR (1.49, 95% CI 1.12–1.99). These results were mainly driven by an increased risk of non‐cardiovascular death, with a weighted event probability at 12 months of 20% (95% CI 14%–28%) and 11% (95% CI 10%–12%) for the statin discontinuation and continuation groups, respectively (Figure 1, HR model‐A 1.56, 95% CI 1.08–2.27; model‐B 1.52, 95% CI 1.06–2.19). There was no evidence for a difference in the rate of fatal or nonfatal cardiovascular events; the weighted 12‐month event probability was 7% (95% CI 4%–13%) for statin discontinuation and 8% (95% CI 7%–9%) for continuation (Figure 1, HR model‐A 1.36, 95% CI .86–2.14; model‐B 1.35, 95% CI .86–2.12). Results from the ‘per‐protocol’ analysis were similar to the main analysis (Table S5). Conclusions were also unchanged in sensitivity analyses (Tables S6–S10). The E‐value for unmeasured confounding was 1.96 for the primary composite outcome; hence, confounders would have to be associated with a 2‐fold increase of the outcome and prevalence in statin discontinuation to account for the result.

**TABLE 2:** Summary of results at 12 months.

Outcome	Statin discontinuation	Statin continuation	Hazard ratio (95% CI)		
	Crude *n* (%) or mean score (SD)^a^	Crude *n* (%) or mean score (SD)^a^	Crude^a^	Model A^b^	Model B^c^
Primary and secondary outcomes					
Death or cardiovascular event	33 (24.8%)	362 (14.3%)	1.83 (1.28 to 2.62)	1.53 (1.14 to 2.06)	1.49 (1.12 to 1.99)
Fatal or nonfatal cardiovascular event	8 (6.0%)	155 (6.1%)	1.04 (.51 to 2.11)	1.36 (.86 to 2.14)	1.35 (.86 to 2.12)
Nonfatal cardiovascular event	8 (6.0%)	135 (5.3%)	1.19 (.58 to 2.43)	1.39 (.85 to 2.26)	1.38 (.85 to 2.25)
Fatal cardiovascular event	0 (0%)	27 (1.1%)	Not estimable	1.00 (.30 to 3.28)	.99 (.30 to 3.26)
Non‐cardiovascular death	26 (19.5%)	223 (8.8%)	2.33 (1.55 to 3.49)	1.56 (1.08 to 2.27)	1.52 (1.06 to 2.19)
Cancer death	12 (9.0%)	72 (2.8%)	3.32 (1.80 to 6.13)	1.91 (.95 to 3.82)	1.71 (.89 to 3.29)
Fall‐related injury or fracture	27 (20.3%)	521 (20.6%)	1.06 (.72 to 1.56)	1.04 (.77 to 1.39)	1.02 (.76 to 1.36)
Barthel index <90	39 (29.3%)	742 (29.3%)	1.29^d^ (.85 to 1.97)	1.13^d^ (.66 to 1.94)	1.10^d^ (.62 to 1.96)
EQ‐5D VAS	62.9 (17.4)	67.0 (18.8)	−4.13^e^ (−7.71 to −.56)	−2.11^e^ (−5.89 to 1.67)	−2.08^e^ (−5.89 to 1.73)
Negative control outcome					
Gastrointestinal disorder	3 (2.3%)	88 (3.5%)	.68 (.22 to 2.15)	1.17 (.64 to 2.13)	1.17 (.63 to 2.14)
Sensitivity analysis: primary outcome including heart failure					
Death or cardiovascular event incl. heart failure	41 (30.8%)	467 (18.4%)	1.80 (1.31 to 2.47)	1.42 (1.10 to 1.83)	1.39 (1.08 to 1.79)
Fatal or nonfatal cardiovascular event incl. heart failure	22 (16.5%)	322 (12.7%)	1.40 (.91 to 2.15)	1.29 (.95 to 1.75)	1.30 (.95 to 1.77)

Abbreviations: CI, confidence interval; SD, standard deviation; VAS, visual analogue scale.

^a^ Pre‐cloned data and unadjusted model.

^b^ Estimated using inverse probability weighting with stabilized weights controlling for randomization group, study site, age, sex, glucose‐lowering drugs, statin intensity, Barthel index, comorbidities (cardiovascular disease, cancer [except skin], chronic respiratory disease, heart failure).

^c^ As Model A, but additionally controlled for the number of falls and weight lost in the previous year.

^d^ Odds ratio (95% CI).

^e^ Mean difference (95% CI).

Results of secondary outcomes did not show evidence for an association between statin discontinuation and fall‐related injuries or fractures, the Barthel Index score, or the EQ‐5D VAS score (Table 2). Similarly, there was no evidence for an association between statin discontinuation and incident gastrointestinal disorders (Table 2).

### 3.3 Subgroup analyses

Subgroup analyses by statin deprescribing for primary and secondary cardiovascular prevention did not provide evidence for a difference in the primary composite outcome or in the cardiovascular events outcome (Table S4). Interestingly, the association between statin discontinuation and non‐cardiovascular death was significantly weaker for secondary cardiovascular prevention (HR 1.23, 95% CI .80–1.91) compared to primary prevention (HR 2.10, 95% CI 1.06–4.17; *p*‐value for interaction = .036). There was also evidence for a difference on the EQ‐5D VAS (*p*‐value for interaction = .044) and weak evidence for a difference in the Barthel Index score (*p*‐value for interaction = .069), with statin discontinuation being associated with lower EQ‐5D VAS and higher odds of Barthel index <90 versus continuation in primary prevention but not in secondary prevention.

## 4 DISCUSSION

In this target trial emulation of statin discontinuation prescribed in multimorbid older patients for primary and secondary cardiovascular prevention, statin discontinuation was associated with a higher risk of the composite outcome of cardiovascular events or all‐cause mortality over 12 months. This excess was mainly due to an increased risk of non‐cardiovascular deaths. In contrast with previous observational studies, there was no clear evidence for an association of statin discontinuation with cardiovascular events in this multimorbid older population. There was also no association with secondary outcomes of incident falls, functional independence status, or health‐related quality of life. These results were not modified by adjusting for the common geriatric syndromes of falls and weight loss.

To our knowledge, this is the first target trial emulation to assess statin discontinuation in an exclusively multimorbid older population.^13^ One small RCT of patients in palliative care did not find a significant difference in cardiovascular events between statin discontinuation and continuation, and quality of life improved marginally in the discontinuation group.^3^ However, the short‐term survival of most patients in the palliative care setting may account for the lack of any survival difference. Our study did not provide clear evidence for an association between statin discontinuation and cardiovascular events (HR 1.35, 95% CI .86–2.12). Although the point estimate was consistent with previous observational studies in older adults (HR range: 1.14–1.32),^8^, ^9^, ^10^ unlike previous studies we found similar absolute cardiovascular risk at 12 months between statin discontinuation (7%) and statin continuation (8%). However, confidence intervals were wide due to the low event rate. Unlike previous studies, we used a ‘clone‐censor‐weighting’ approach to address immortal‐time bias which avoids limitations of the landmark and time‐varying exposure models and upholds target trial emulation principles.^14^ Results from our study are not directly comparable to studies of older patients initiating statins due to different patient populations (statin‐naïve versus statin users) and potential legacy effects of statins.^23^

In the present cohort of multimorbid older adults with polypharmacy, statin discontinuation over 12 months' follow‐up was uncommon (5% of patients). A reason for the low discontinuation rate may be the high prevalence of established cardiovascular disease in this population (>70%, Table 1), as general practitioners may be less inclined to discontinue statins for secondary prevention even in the presence of side effects and frailty.^24^ Other potential reasons relating to deprescribing choices among both healthcare professionals and patients may include lack of evidence and guidelines on deprescribing statins, fear of health deterioration and patient preferences.^24^ Indeed, benefits of statins may extend cardiovascular risk reduction, reducing mortality even in lean older individuals with liver fibrosis.^25^ Whether such effects persist with multimorbidity and polypharmacy remains unclear. Notably, drug–drug interactions are common in polypharmacy (58% of participants had an interaction in the present study),^26^ including interactions with statins,^27^ and multiple severe interactions may increase mortality.^28^

While the target trial emulation framework helps to reduce certain avoidable biases (e.g., prevalent user and immortal time biases), confounding bias remains a major concern.^29^ We observed a large increased risk of non‐cardiovascular death with statin discontinuation that is both clinically and pathophysiologically implausible,^30^ indicating probable confounding bias. Controlling for reduced functional independence (Barthel Index score), frequency of past falls and weight loss as common geriatric syndromes in our analyses did not eliminate this association. Poor overall health and frailty have been suggested as common confounders.^8^ Perceived short life expectancy may confound results as it was associated with primary care physicians' advice to stop statins in older patients (OR of 50.7),^24^ exceeding the E‐value of 2 needed to explain results.^22^ However, a sensitivity analysis excluding patients with cancer at baseline did not change results. Since similar issues with confounding influences have been observed previously,^8^, ^29^ future studies should carefully consider potential biases, particularly by examining non‐cardiovascular mortality. Results from ongoing RCTs are needed to firmly establish the benefit–risk of statin discontinuation.^6^

### 4.1 Strengths and Limitations

Strengths of this study were (i) the high‐quality data on a well‐characterized cohort of multimorbid older adults from four European countries participating in the OPERAM trial, and (ii) the rigorous application of target trial emulation principles, including the use of the ‘clone‐censor‐weight’ method which has not been applied in previous studies on statin discontinuation. Importantly, this study is the first to assess statin discontinuation specifically in a multimorbid population.

Our study also has limitations. Residual confounding remained despite controlling for geriatric syndromes. One possible confounder might be perceived short life expectancy, which we could not sufficiently adjust for due to a lack of adequate variables. The reason for statin discontinuation could have provided useful information on confounding by indication, but it was only available for 38% of patients. Additionally, frailty was not explicitly assessed in the OPERAM study. Moreover, the OPERAM study was limited to a one‐year follow‐up and relatively few statin discontinuations (*n* = 133). While effects on cardiovascular events may appear at longer follow‐up, we observed no divergence of cumulative incidence curves between groups. Further, medication use data were based on various sources, including patient report, which may have introduced exposure misclassification due to imprecise reporting of medication start and end dates or non‐adherence. However, adherence was notably high in the OPERAM study.^15^ As participants accepted to be part of a clinical trial, they may differ from routine practice, but it allows for systematic data collection. We acknowledge that the OPERAM trial was not designed to adjudicate deaths resulting from drug–drug interactions, including those of statins.^15^

## 5 CONCLUSION

In this first target trial emulation of multimorbid older adults with polypharmacy enrolled during hospitalization, statin discontinuation was associated with a higher risk of the composite outcome of cardiovascular events or all‐cause mortality. However, non‐cardiovascular mortality was the main driver of the increased risk, suggesting residual confounding by indication. These increased risks were not modified by adjusting for common geriatric syndromes. In contrast to some previous retrospective cohort studies, there was no clear evidence of increased cardiovascular events arising from statin discontinuation. Our study highlights that only RCTs can clarify the safety of statin discontinuation in multimorbid older adults.

## AUTHOR CONTRIBUTIONS

Concept and design: Rodondi, Del Giovane, Baggio, Baretella, Aponte Ribero. Acquisition, analysis, or interpretation of data: All authors. Drafting of the manuscript: Aponte Ribero. Critical revision of the manuscript for important intellectual content: All authors. Statistical analysis: Aponte Ribero, Baggio. Obtained funding: Rodondi, Baretella. Supervision: Rodondi, Baggio.

## FUNDING INFORMATION

This work is part of the project ‘OPERAM: OPtimising thERapy to prevent Avoidable hospital admissions in Multimorbid Older Patients’ supported by the European Union's Horizon 2020 research and innovation program under the grant agreement No 634238, and by the Swiss State Secretariat for Education, Research and Innovation (SERI) under contract number 15.0137 and of the project ‘Discontinuing Statins in Multimorbid Older Adults without Cardiovascular Disease (STREAM)’ supported by the Swiss National Science Foundation (SNSF) under the grant agreement No IICT 33IC30‐193052 (to Prof. Rodondi). The opinions expressed and arguments employed herein are those of the authors and do not necessarily reflect the official views of the European Commission and the Swiss government. VAR was supported by the Swiss National Scientific Foundation (grant number 325130_204361/1). OB obtained a Protected Research Time (PRT) Grant from the University of Bern for this project.

## CONFLICT OF INTEREST STATEMENT

None.

## Supporting information


Appendix S1.

## Data Availability

Data for this study will be made available to others in the scientific community upon request after publication. Data will be made available for scientific purposes for researchers whose proposed use of the data has been approved by a publication committee.
