# Supplementary material for: Target trial emulation of statin discontinuation in multimorbid older adults with polypharmacy
Source: Eur J Clin Invest. 2025 Sep 30;56(1):e70126. doi: 10.1111/eci.70126 (PMC12817241; doi:10.1111/eci.70126)
Supplement: Supplementary file 1 — Appendix S1. [file ECI-56-e70126-s001.docx]

**Supplementary Appendix**

Supplement to: Target Trial Emulation of Statin Discontinuation in Multimorbid Older Adults with Polypharmacy

Contents

[Table S1. ICD-10 codes of baseline comorbidities and outcomes 2](#_Toc207008784)

[Table S2. Specification of the target trial emulation of statin discontinuation using OPERAM data 3](#_Toc207008785)

[Table S3. Number of deaths by cause 4](#_Toc207008786)

[Table S4. Results by primary and secondary prevention 5](#_Toc207008787)

[Table S5. Results at 12 months in the per-protocol analyses 7](#_Toc207008788)

[Table S6. Results at 12 months of analyses without a grace period 8](#_Toc207008789)

[Table S7. Results at 12 months with a grace period of 3 months (90 days) 9](#_Toc207008790)

[Table S8. Results at 12 months with updated cancer information^a^ 10](#_Toc207008791)

[Table S9. Results at 12 months using other Barthel Index cutoff scores 10](#_Toc207008792)

[Table S10. Results at 12 months for participants without cancer at baseline 11](#_Toc207008793)

[Figure S1. Participant flow chart 12](#_Toc207008794)

[Figure S2. Study design 13](#_Toc207008795)

[Figure S3. Directed acyclic graph (DAG) 14](#_Toc207008796)

# Table S1. ICD-10 codes of baseline comorbidities and outcomes

|  | **ICD-10 codes** |
| --- | --- |
| **Baseline conditions** |  |
| Cardiovascular disease | G45, I20–25, I63–65, I70-71, I73.1, I73.8, I73.9, I77.1, I79.0, I79.2, K55, Z95.1, Z95.5, Z95.8, Z95.9 |
| Heart failure | I50 |
| Chronic respiratory disease | J00-J99, Q31-34, R05-06, R91 |
| Cancer (except skin) | C00–C26, C30–C34, C37-C41, C43, C45–C58, C60–C76, C77–C80, C81–C85, C88, C90–C97 |
| **Outcomes** |  |
| Cardiovascular disease | G45, I20-22, I24, I61, I63–67, I70–71, I73.1, I73.8, I73.9, I74, I77.1, I79.2, K55.0, K55.1, K55.8, K55.9 |
| Heart failure | I50 |

Abbreviations: ICD-10, International Classification of Diseases, Tenth Revision

# Table S2. Specification of the target trial emulation of statin discontinuation using OPERAM data

| **Protocol component** | **Target trial specification** | **Target trial emulation** |
| --- | --- | --- |
| Eligibility criteria | Age ≥70 years, multimorbidity with ≥3 chronic diseases of ≥6 months duration, polypharmacy with ≥5 chronic medications including a statin at enrolment | Same as for the target trial |
| Treatment strategies | 1. Statin continuation with therapy existing at baseline 2. Statin discontinuation at baseline | Same as for the target trial, but with a grace period.  Three trials will be emulated with baseline at hospital discharge, 2-months follow-up and 6-months follow-up. |
| Treatment assignment | Individuals will be randomly assigned to a strategy at baseline and they and their treating physicians will be aware of the strategy assigned | We will classify individuals according to the strategy compatible with their data at baseline and will attempt to emulate randomization by adjusting for baseline confounders |
| Follow-up | Starts at baseline and ends at the date of the outcome, loss to follow-up, or end of the one-year follow-up. | Same as for the target trial |
| Outcomes | Primary: composite of cardiovascular events (myocardial infarction, stroke, transient ischemic attack, peripheral vascular disease) and all-cause mortality  Secondary: quality of life (EQ-5D VAS), activities of daily living (Barthel index), and falls and fractures | Same as for the target trial |
| Causal contrasts | Intention-to-treat effect  Per-protocol effect | Observational analogues of intention-to-treat and per protocol effect |
| Statistical analysis | Intention-to-treat analysis  Per-protocol analysis: censor participants if and when deviating from their assigned treatment strategy and application of inverse probability weights to adjust for pre- and post-baseline prognostic factors associated with adherence  Subgroup analyses by primary *versus* secondary cardiovascular prevention | Modified intention-to-treat analysis, in which patients are censored when deviating from the assigned strategy by the end of the grace period (static treatment assumed thereafter), and per-protocol analyses with sequential emulation including adjustment for baseline covariates  Same exploratory subgroup analyses |

Abbreviations: VAS, visual analogue scale

# Table S3. Number of deaths by cause

| **Causes of death** | **Number of deaths (%)**  **Total N=281** |
| --- | --- |
| Acute coronary syndrome | 14 (5.0%) |
| Bleeding | 4 (1.4%) |
| Cancer | 86 (30.6%) |
| Heart failure | 35 (12.5%) |
| Infection/sepsis/septic shock | 31 (11.0%) |
| Ischemic stroke | 13 (4.6%) |
| Pulmonary embolism | 5 (1.8%) |
| Renal failure | 8 (2.8%) |
| Sudden death without known aetiology | 1 (0.4%) |
| Other | 84 (29.9%) |

# Table S4. Results by primary and secondary prevention

|  | **Primary prevention (N=731)** | | | | **Secondary prevention (N=1,937)** | | | | **Interaction p-value** |
| --- | --- | --- | --- | --- | --- | --- | --- | --- | --- |
| Outcome | Statin discontinuation | Statin continuation | Hazard ratio (95% CI) | | Statin discontinuation | Statin continuation | Hazard ratio (95% CI) | |  |
|  | Crude n (%) or mean score (SD)^a^ | Crude n (%) or mean score (SD)^a^ | Model A^b^ | Model B^c^ | Crude n (%) or mean score (SD)^a^ | Crude n (%) or mean score (SD)^a^ | Model A^b^ | Model B^c^ |  |
| N | 57 | 674 |  |  | 76 | 1,861 |  |  |  |
| **Primary and secondary outcomes** | | | | |  |  |  |  |  |
| Death or cardiovascular event | 17 (25.4%) | 53 (7.9%) | 1.89  (1.03 to 3.47) | 1.76  (0.95 to 3.25) | 45 (34.1%) | 309 (16.7%) | 1.38  (0.99 to 1.92) | 1.35  (0.97 to 1.87) | 0.219 |
| Fatal or nonfatal cardiovascular event | 2 (3.0%) | 18 (2.7%) | 0.68  (0.15 to 3.04) | 0.65  (0.14 to 3.07) | 21 (15.9%) | 137 (7.4%) | 1.41  (0.88 to 2.27) | 1.41  (0.88 to 2.27) | 0.458 |
| Nonfatal cardiovascular event | 2 (3.5%) | 18 (2.7%) | 0.70  (0.16 to 3.14) | 0.67  (0.14 to 3.16) | 6 (7.9%) | 118 (6.4%) | 1.45  (0.86 to 2.42) | 1.45  (0.87 to 2.42) | 0.464 |
| Fatal cardiovascular event | 0 (0%) | 1 (0.1%) | Not estimable | Not estimable | 0 (0%) | 26 (1.4%) | 1.07  (0.32 to 3.59) | 1.08  (0.32 to 3.62) | Not estimable |
| Non-cardiovascular death | 15 (22.4%) | 37 (5.5%) | 2.28  (1.14 to 4.56) | 2.10  (1.06 to 4.17) | 25 (18.9%) | 189 (10.2%) | 1.29  (0.83 to 2.01) | 1.23  (0.80 to 1.91) | 0.036 |
| Cancer death | 5 (7.5%) | 13 (1.9%) | 2.32  (0.60 to 8.92) | 2.11  (0.58 to 7.66) | 8 (6.1%) | 61 (3.3%) | 1.55  (0.68 to 3.55) | 1.28  (0.58 to 2.82) | 0.228 |
| Fall-related injury or fracture | 17 (25.4%) | 125 (18.6%) | 1.13  (0.65 to 1.97) | 1.12  (0.64 to 1.97) | 42 (31.8%) | 400 (21.6%) | 1.05  (0.75 to 1.46) | 1.03  (0.74 to 1.44) | 0.809 |
| Barthel index <90 | 18 (26.9%) | 211 (31.4%) | 1.88^d^  (0.80 to 4.42) | 1.91^d^  (0.82 to 4.46) | 20 (15.2%) | 525 (28.4%) | 0.95^d^  (0.44 to 2.03) | 0.90^d^  (0.40 to 2.02) | 0.069 |
| EQ-5D VAS | 59.3 (18.5) | 68.2 (18.1) | -7.49^e^  (-14.87 to -0.12) | -7.45^e^  (-15.03 to 0.14) | 65.6 (16.3) | 66.6 (19.2) | 0.22^e^  (-4.06 to 4.50) | 0.26^e^  (-4.10 to 4.62) | 0.044 |
| **Negative control outcome** | | |  | |  |  |  |  |  |
| Gastrointestinal disorder | 4 (6.0%) | 24 (3.6%) | 0.89  (0.29 to 2.80) | 0.79  (0.25 to 2.54) | 9 (6.8%) | 64 (3.5%) | 1.27  (0.61 to 2.61) | 1.27  (0.61 to 2.62) | 0.876 |

Primary and secondary cardiovascular prevention defined according to ICD-10 codes listed in Table S1. ^a^ Pre-cloned data and unadjusted; ^b^ Estimated using inverse probability weighting with stabilized weights controlling for randomization group, study site, age, sex, glucose-lowering drugs, statin intensity, Barthel index, comorbidities (cardiovascular disease, cancer [except skin], chronic respiratory disease, heart failure); ^c^ As Model A, but additionally controlled for number of falls and weight lost in the previous year; ^d^ Odds ratio (95% CI); ^e^ Mean difference (95% CI)

Abbreviations: CI, confidence interval; SD, standard deviation; VAS, visual analogue scale

# Table S5. Results at 12 months in the per-protocol analyses

| Outcome | Hazard ratio (95% CI) | | |
| --- | --- | --- | --- |
|  | Model A^a^ | | Model B^b^ |
| **Primary and secondary outcomes** | | | |
| Death or cardiovascular event | 1.67 (1.23 to 2.27) | | 1.61 (1.20 to 2.17) |
| Fatal or nonfatal cardiovascular event | 1.42 (0.89 to 2.29) | | 1.43 (0.89 to 2.30) |
| Non-cardiovascular death | 1.81 (1.22 to 2.68) | | 1.73 (1.19 to 2.52) |
| Fall-related injury or fracture | 1.09 (0.81 to 1.46) | | 1.07 (0.80 to 1.44) |
| Barthel index <90 | 0.77 (0.35 to 1.72)^c^ | | 0.71 (0.29 to 1.71)^c^ |
| EQ-5D VAS | 3.06 (-1.85 to 7.97)^d^ | | 2.25 (-2.73 to 7.22)^d^ |
| **Negative control outcomes** | |  |  |
| Gastrointestinal disorder | 1.24 (0.67 to 2.29) | | 1.24 (0.67 to 2.32) |

^a^ Estimated using inverse probability weighting with stabilized weights controlling for randomization group, study site, age, sex, glucose-lowering drugs, statin intensity, Barthel index, comorbidities (cardiovascular disease, cancer [except skin], chronic respiratory disease, heart failure); ^b^ As Model A, but additionally controlled for number of falls and weight lost in the previous year; ^c^ Odds ratio (95% CI); ^d^ Mean difference (95% CI)

Abbreviations: CI, confidence interval; VAS, visual analogue scale

# Table S6. Results at 12 months of analyses without a grace period

| Outcome | Hazard ratio (95% CI) | | |
| --- | --- | --- | --- |
|  | Model A^a^ | | Model B^b^ |
| **Primary and secondary outcomes** | | | |
| Death or cardiovascular event | 1.77 (1.03 to 3.04) | | 1.77 (1.02 to 3.05) |
| Fatal or nonfatal cardiovascular event | 1.08 (0.47 to 2.49) | | 1.06 (0.45 to 2.49) |
| Non-cardiovascular death | 2.21 (1.16 to 4.21) | | 2.27 (1.19 to 4.35) |
| Fall-related injury or fracture | 1.34 (0.60 to 2.99) | | 1.45 (0.66 to 3.22) |
| Barthel index <90 | 1.21 (0.56 to 2.61)^c^ | | 1.33 (0.60 to 2.94)^c^ |
| EQ-5D VAS | -3.32 (-10.12 to 3.47)^d^ | | -3.06 (-9.61 to 3.50)^d^ |
| **Negative control outcomes** | |  |  |
| Gastrointestinal disorder | 0.35 (0.05 to 2.51) | | 0.33 (0.04 to 2.43) |

^a^ Estimated using inverse probability weighting with stabilized weights controlling for randomization group, study site, age, sex, glucose-lowering drugs, statin intensity, Barthel index, comorbidities (cardiovascular disease, cancer [except skin], chronic respiratory disease, heart failure); ^b^ As Model A, but additionally controlled for number of falls and weight lost in the previous year; ^c^ Odds ratio (95% CI); ^d^ Mean difference (95% CI)

Abbreviations: CI, confidence interval; VAS, visual analogue scale

# Table S7. Results at 12 months with a grace period of 3 months (90 days)

| Outcome | Hazard ratio (95% CI) | | |
| --- | --- | --- | --- |
|  | Model A^a^ | | Model B^b^ |
| **Primary and secondary outcomes** | | | |
| Death or cardiovascular event | 1.34 (1.06 to 1.68) | | 1.32 (1.05 to 1.66) |
| Fatal or nonfatal cardiovascular event | 1.13 (0.79 to 1.62) | | 1.12 (0.78 to 1.61) |
| Non-cardiovascular death | 1.34 (1.01 to 1.79) | | 1.33 (1.00 to 1.78) |
| Fall-related injury or fracture | 1.03 (0.84 to 1.27) | | 1.03 (0.84 to 1.27) |
| Barthel index <90 | 0.85 (0.52 to 1.40)^c^ | | 0.84 (0.50 to 1.40)^c^ |
| EQ-5D VAS | -0.26 (-3.68 to 3.15)^d^ | | -0.25 (-3.68 to 3.19)^d^ |
| **Negative control outcomes** | |  |  |
| Gastrointestinal disorder | 1.09 (0.68 to 1.74) | | 1.09 (0.68 to 1.75) |

^a^ Estimated using inverse probability weighting with stabilized weights controlling for randomization group, study site, age, sex, glucose-lowering drugs, statin intensity, Barthel index, comorbidities (cardiovascular disease, cancer [except skin], chronic respiratory disease, heart failure); ^b^ As Model A, but additionally controlled for number of falls and weight lost in the previous year; ^c^ Odds ratio (95% CI); ^d^ Mean difference (95% CI)

Abbreviations: CI, confidence interval; VAS, visual analogue scale

# Table S8. Results at 12 months with updated cancer information^a^

| Outcome | Hazard ratio (95% CI) | | |
| --- | --- | --- | --- |
|  | Model A^b^ | | Model B^c^ |
| **Primary and secondary outcomes** | | | |
| Death or cardiovascular event | 1.51 (1.14 to 2.01) | | 1.49 (1.12 to 1.97) |
| Fatal or nonfatal cardiovascular event | 1.35 (0.86 to 2.12) | | 1.33 (0.85 to 2.08) |
| Non-cardiovascular death | 1.56 (1.10 to 2.22) | | 1.56 (1.10 to 2.21) |
| Cancer death | 1.91 (1.02 to 3.59) | | 1.88 (1.01 to 3.52) |
| Fall-related injury or fracture | 1.02 (0.77 to 1.34) | | 1.00 (0.76 to 1.32) |
| Barthel index <90 | 1.23 (0.74 to 2.05)^d^ | | 1.23 (0.73 to 2.07)^d^ |
| EQ-5D VAS | -3.11 (-6.71 to 0.49)^e^ | | -3.20 (-6.78 to 0.39)^e^ |
| **Negative control outcomes** | |  |  |
| Gastrointestinal disorder | 1.16 (0.64 to 2.13) | | 1.15 (0.63 to 2.11) |

^a^ New cancer hospitalisations occurred for 6 persons (1 by the 2-month follow-up, 5 by the 6-months follow-up), all in the statin continuation group. ^b^ Estimated using inverse probability weighting with stabilized weights controlling for randomization group, study site, age, sex, glucose-lowering drugs, statin intensity, Barthel index, comorbidities (cardiovascular disease, cancer [except skin], chronic respiratory disease, heart failure); ^c^ As Model A, but additionally controlled for number of falls and weight lost in the previous year; ^d^ Odds ratio (95% CI); ^e^ Mean difference (95% CI)

Abbreviations: CI, confidence interval; VAS, visual analogue scale

# Table S9. Results at 12 months using other Barthel Index cutoff scores

| Outcome | Hazard ratio (95% CI) | |
| --- | --- | --- |
|  | Model A^b^ | Model B^c^ |
| **Barthel Index cutoff ≤60 vs. >60** | | |
| Death or cardiovascular event | 1.46 (1.09 to 1.95) | 1.42 (1.07 to 1.90) |
| Fatal or nonfatal cardiovascular event | 1.35 (0.86 to 2.14) | 1.34 (0.85 to 2.12) |
| Non-cardiovascular death | 1.47 (1.01 to 2.13) | 1.43 (1.00 to 2.06) |
| **Barthel Index cutoff ≤20 vs. >20** |  |  |
| Death or cardiovascular event | 1.51 (1.13 to 2.02) | 1.47 (1.10 to 1.96) |
| Fatal or nonfatal cardiovascular event | 1.35 (0.88 to 2.18) | 1.37 (0.87 to 2.16) |
| Non-cardiovascular death | 1.54 (1.06 to 2.23) | 1.49 (1.04 to 2.14) |

# Table S10. Results at 12 months for participants without cancer at baseline

| Outcome | Statin discontinuation | Statin continuation | Hazard ratio (95% CI) | |
| --- | --- | --- | --- | --- |
|  | Crude n (%) or mean score (SD)^a^ | Crude n (%) or mean score (SD)^a^ | Model A^b^ | Model B^c^ |
| N | 94 | 1857 |  |  |
| **Primary and secondary outcomes** | | | | |
| Death or cardiovascular event | 18 (19.1%) | 243 (13.1%) | 1.48  (1.05 to 2.09) | 1.48  (1.05 to 2.09) |
| Fatal or nonfatal cardiovascular event | 5 (5.3%) | 125 (6.7%) | 1.23  (0.73 to 2.05) | 1.22  (0.73 to 2.04) |
| Nonfatal cardiovascular event | 5 (5.3%) | 105 (5.7%) | 1.24  (0.71 to 2.17) | 1.23  (0.70 to 2.17) |
| Fatal cardiovascular event | 0 (0%) | 26 (1.4%) | 0.98  (0.30 to 3.25) | 1.01  (0.30 to 3.35) |
| Non-cardiovascular death | 13 (13.8%) | 132 (7.1%) | 1.62  (1.03 to 2.56) | 1.64  (1.03 to 2.59) |
| Cancer death | 3 (3.2%) | 22 (1.2%) | 1.47  (0.40 to 5.40) | 1.47  (0.39 to 5.51) |
| Fall-related injury or fracture | 20 (21.3%) | 388 (20.9%) | 1.03  (0.73 to 1.46) | 0.98  (0.70 to 1.37) |
| Barthel index <90 | 41 (43.6%) | 982 (52.9%) | 1.01^d^  (0.53 to 1.94) | 0.98^d^  (0.48 to 1.97) |
| EQ-5D VAS | 64.1 (17.0) | 67.0 (19.5) | -1.86^e^  (-6.10 to 2.39) | -1.32^e^  (-5.71 to 3.08) |
| **Negative control outcome** | | |  | |
| Gastrointestinal disorder | 3 (3.2%) | 64 (3.4%) | 1.44  (0.77 to 2.71) | 1.44  (0.76 to 2.71) |

Cancer was defined according to ICD-10 codes listed in Table S1. ^a^ Pre-cloned data and unadjusted; ^b^ Estimated using inverse probability weighting with stabilized weights controlling for randomization group, study site, age, sex, glucose-lowering drugs, statin intensity, Barthel index, comorbidities (cardiovascular disease, cancer [except skin], chronic respiratory disease, heart failure); ^c^ As Model A, but additionally controlled for number of falls and weight lost in the previous year; ^d^ Odds ratio (95% CI); ^e^ Mean difference (95% CI)

Abbreviations: CI, confidence interval; SD, standard deviation; VAS, visual analogue scale

# Figure S1. Participant flow chart


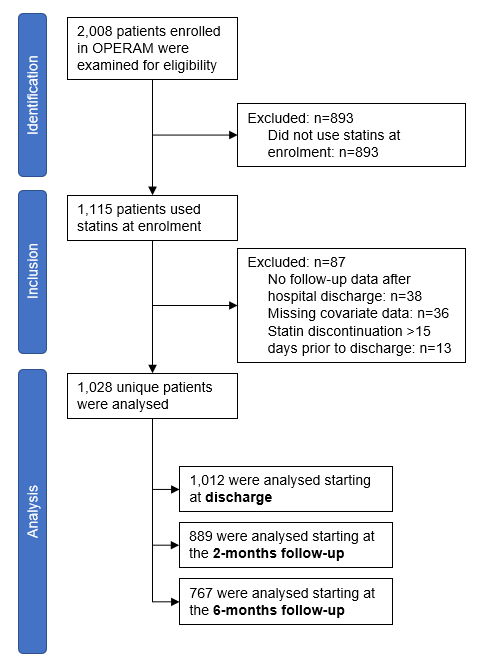


# Figure S2. Study design


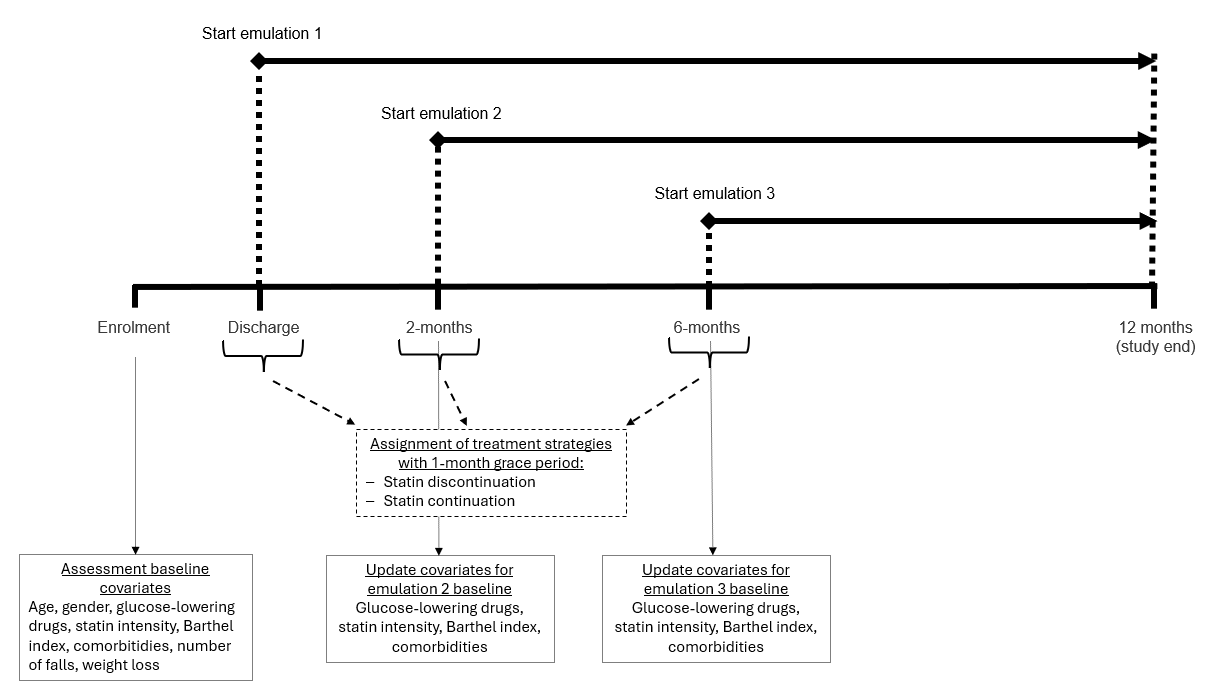


# Figure S3. Directed acyclic graph (DAG)


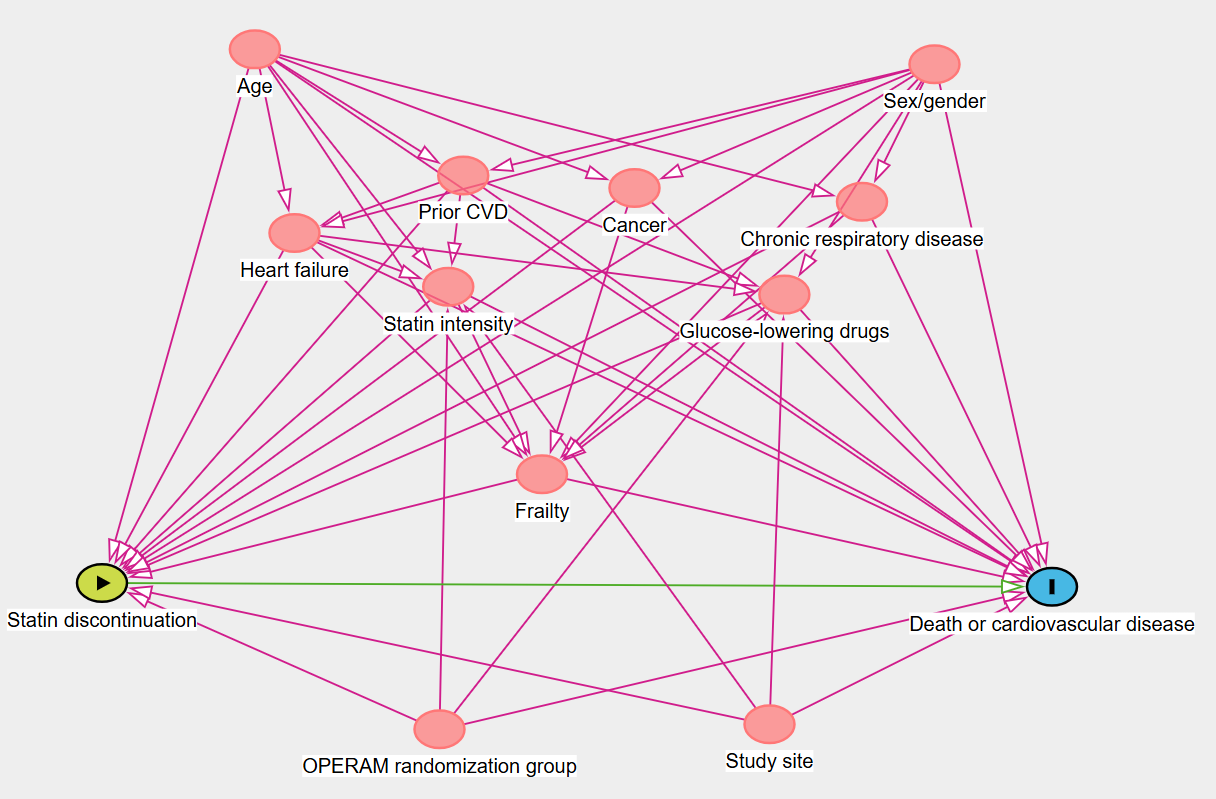


The DAG illustrates the assumed causal structure between statin discontinuation (exposure, green-shaded circle) and death or cardiovascular disease (outcome, blue-shaded circle) including potential confounders of this relationship (red-shaded circles). The DAG was drawn on <https://www.dagittyCance.net/dags.html>
